# Un-Certainty as a Pragmatic Resource for Psychiatric Argumentation: a Diachronical and Diatextual Approach

**DOI:** 10.1007/s12124-020-09568-7

**Published:** 2020-07-18

**Authors:** Rosa Scardigno, Giuseppe Mininni

**Affiliations:** Department of Educational Sciences, Psychology, Communication, University of Bari Aldo Moro, Via Crisanzio, 42, 70121 Bari, Italy

**Keywords:** Un-certainty, Psychiatry, Rhetoric, Mitigation, Authorship

## Abstract

Psychiatry is the science that aims to propose plausible theories in the description and explanation of “body-mind” pathologies. Since also the modern institution of science produces a type of discourse aimed at reducing human *insecuritas* through a progressive falsification of conjectures on how things are actually, it seems very important to monitor the discursive construction of un-certainty about an extremely elusive object such as the abnormality of psychic functioning. In the light of this, the present paper aims to identify what changes are traceable in the argumentative structure of un-certainty in the psychiatric scientific communication by the British Journal of Psychiatry in its life span as well as how the construction of socially “credible” authorship profiles evolves. The randomly-selected 90 articles from the 160 years of the BJP life cycle were analyzed through various interpretative apparatuses, by practicing both bottom-up and top-down approaches. Indeed, in the perspective of cultural and discursive psychology, un-certainty is a multidimensional discursive construction which is not attributable to the psycho-linguistic level of the utterance in its entirety, but to the meta-pragmatic dimension of enunciation. The identified rhetoric, which collects the groupings of enunciative profiles, sees the researcher evolving between the explorer’s attempts, the investigator’s inquiries and the critical rigor of the technician.

## Introduction

Psychiatry is a branch of medical knowledge that, after a long gestation (Foucault 1961), has found legitimacy and relative autonomy only in the last two centuries. Its specificity consists in facing the specific type of pathologies that affect the psychophysical unity of the human being, i.e. its “body-mind” being. Progressively modern (Western) scholars have developed a system of knowledge, practices and techniques to address the problems posed by behaviours considered as “anomalous” and the inexplicable suffering of some people labelled as “alienated”, “crazy” and “mentally-ill”. From its origins, psychiatry appears to be inspired by a dominant taxonomic tendency. From the primitive classifications by Kraepelin to the DSM-5, the cognitive tension appears to be aimed at setting up interpretive frameworks capable of grasping the (elusive) phenomenology of mental illness.

The British Journal of Psychiatry (BJP) represents an important site of the scientific debate that has been the background to the genesis of a growing body of knowledge and therapeutic practices related to psychiatry. Its first issue was published in 1858 and for several decades the expression “The Journal of Mental Science” appeared as a subtitle in the title page. For over a century and a half, BJP has provided scholars with the opportunity to circulate, among an ever wider community, the knowledge that they were gradually developing on psychic pathologies and on the most suitable ways to cure them. Consequently, the BJP represents a very important observatory to monitor the discursive construction of un-certainty about an extremely elusive object such as the abnormality of psychic functioning, which is significantly affected by the historical and cultural conditions in which it is realized. The corpus of articles analyzed here – i.e. a sample of texts published in the BJP from 1858 to 2014 – makes it possible to test some hypotheses on the complex enunciative meaning stratification that generates the specific “tone” of psychiatric discourse.

The scientific article, being a text aimed at informing specialized contents through the use of technical terminology, assumes a predominantly argumentative and expositive plot, with a persuasive and applicative function. Generally, it focuses on the unresolved problems of science or on those issues that still have aspects to investigate. Therefore, modulation of un-certainty is a crucial aspect, since the way in which the same information is communicated can result in opposite applicative outcomes. For example, national health policies are constructed on the basis of how results of biomedical research are communicated; clinical practice follows the same logic in the adoption of new therapies, in prevention and diagnosis. Indeed, being a means to promote scholars onto a professional and academic level, the authors often resort to an attitude of “caution”, by using discursive tools designed to show modesty, honesty and prudence, to obtain acceptance of results, avoid potential waste and create a space for discussion among experts (Morales 2010; Swales 1990).

## The “Hand-to-Hand Fight” with the Mind

The modern institution of science makes a kind of discourse aimed at reducing human insecuritas (Semerari 2005) available through a progressive falsification of conjectures on how things are actually in the various areas of world experience. Among the reassuring techniques invented by man – religion, art, philosophy and science –, the great tradition of Western thought appears animated by the obsession of (un)certainty, which has inspired multiple theories concerning the ways and limits of knowledge as form of control of human beings over the world (McBurney and Parsons 2002).

Psychiatry is the science that aims to propose plausible theories in the description and explanation of “body-mind” pathologies (Martin 2000; Panksepp 2004), i.e. of the psycho-physical unity inherent to the human condition. The historical and theoretical comparison between the disciplinary structures allows us to distinguish psychiatry from clinical psychology (Gabbard 2014). While the latter is focused on the possibilities of “healing the mind with the resources of the mind” – systems of beliefs, relocations of relationships, narrative modules, etc. –, the former proposes models of analysis and intervention that are mainly based on bodily functionality. Nevertheless, both disciplines evoke a broader horizon of reference – i.e. medical psychology (Donnelly 1983) –, which focuses on the contribution that the scientific knowledge of the mind can provide in the multiple and difficult practices of medicine (from the communication of diagnosis to the management of therapy).

The evident predominance of neuroscience in the current cultural panorama makes the implantation of psychiatric knowledge in the treatment of psychopathologies particularly salient. Indeed, from the vague and stigmatizing concept of “madness” to the subtle distinctions of the classification system proposed by the DSM-5, psychiatric knowledge is characterized by a specific aspiration to overcome Cartesian dualism, marking the organic “body-mind” unity. This tension also reverberates in the forms and models of discursive construction of un-certainty (Scardigno et al. 2017) with respect to its theoretical and applicative proposals (both cognitive and therapeutic). Psychiatric treatment is legitimized mostly on a set of knowledge that supports the expectation of being able to “support the mind” by intervening “on the body”. From electroshock to psychotropic drugs, the interpretative and procedural resources that psychiatry relies on highlight the need to re-establish a balance in bodily function in order to promote psychological well-being.

It is therefore reasonable to expect that the epistemic weaving of psychiatric knowledge is also nourished by the complex dynamics that can be traced back to the almost random nature of the body-mind system.

## The (Discursive) Construction of Un-Certainty

In the history of Western philosophy, the theme of certainty is firmly intertwined with the possibility of identifying the truth, in a continuous and reciprocal appeal. From the millennial philosophical elaboration on the theme of un-certainty, some conceptual junctions are more pertinent to the aims of our work, such as Locke’s (1690) need to consider certainty in terms of its linguistic expression and the lucid analysis provided by Wittgenstein, aimed at demonstrating that “certainty is, so to speak, a tone in which the state of things is acknowledged; but the tone does not mean that one is right” (1969, 8, our translation).

In this cultural horizon a psycholinguistic perspective aims to penetrate all the practices of sense-making, from the dynamics of common discourse to the scientific communication, where it can be particularly effective for comparing argumentative styles. Indeed, un-certainty has its own specific language (Clark 1990; Teigen 1988) which covers the whole range of the linguistic system, from the lexical-grammatical to the textual-stylistic axis.

In any form of discursive life – from the legal world (Mininni et al. 2014b) to the religious sphere (Scardigno and Mininni 2014) – the philosophical distinction between subjective certainty (“certitude”, or the feeling of security that people have in relation to some certain knowledge that they consider to be true and stable) and objective (“certainty”, i.e. the reference to the actual reasons that establish the reliability and the truth of the knowledge) is echoed in (psycho) linguistics by the debate on the difference between “evidence” and “epistemicity” (Wesson and Pulford 2009; Zuczkowski et al. 2017). While evidence indexes focus on the source of information (I saw / remember / know ...), indexes of epistemicity (e.g. adverbs such as ‘sure’, ‘undoubtedly’, ‘perhaps’, ‘probably’) reveal the attitude of the speaker with respect to the reliability of information (González 2005), the judgment on the probability of propositions (Cappelli 2007) or the commitment to the truth of a message (De Haan 1999).

A very important macro-category of expressions of un-certainty is the mode, which can take place at various levels, from positioning in the lexical area (“certainty”, “plausibility”, “probability”, “doubt”) and the related verbs that enunciate the statement (“I have the evidence”, “It is expected”, “It is uncertain”) to the quantitative and qualitative support offered by the adjectives that accompany a certain positioning, from the syntactical form – assertion (positive and negative), question, etc. – syntagmatic structure (indicative vs conditional, present vs. future, etc.), up to rhetorical strategies (accumulation, irony, metalinguistic comment). The expressions of modal un-certainty (Halliday 1970) provide the enunciator with a wide range of resources which modulate the degree of commitment and, as a consequence, intentional transparency for the respect of the “trust contract” with their potential readers and assumption of validity of what s/he says. In its most “certain” form represented by the verb servile “duty”, this modulation exhibits a claim of certainty as it draws from the illo-perlocutor force from the “ethics of discourse” (Habermas 1993).

Our inquiry into un-certainty is placed within a theoretical and conceptual framework that is compatible with the perspective of cultural and discursive psychology (Manuti et al. 2017). Namely, the basic assumption on which our hypothesis is based is that un-certainty is a multidimensional discursive construction, attributable in its entirety not to the psycho-linguistic level of the utterance, but to the meta-pragmatic dimension of enunciation (Caffi 2007). As an effect of special “psychodiscursive practices” (Wetherell 2008), un-certainty corresponds to a fundamental configuration of the psyche that expresses a sense potential in its tension between stability and instability, between figure and background, between “a safe base” and “a need for exploration”.

In our investigation we do not make use of the many heterogeneous tools that the various disciplines (from psychology to semiotics, from ancient rhetoric to modern pragmalinguistics) have elaborated to account for the modulation located in the enunciation (Caffi 2007). Since un-certainty is a tonal quality of discourse, to be captured at best, it should be framed with an approach that is able not only to integrate multiple procedures of analysis, but above all to grasp its proteiform nature in a holistic interpretation.

Un-certainty is a regime of “modality” in the discursive construction and such a modulation can make use of multiple textual and contextual resources, which also vary according to the language and to the reference culture. Indeed, the modulation of un-certainty is practicable with the following resources:

- prosodic ones (intensity of voice, intonation, etc.);
- lexical ones, such as verbs (e.g. “I suppose”), names (e.g. “probability”, “usual”, etc.), evaluative adjectives (e.g. “big”, “tall”, “wide”, etc.);
- grammatical ones, such as the modes and timing of verbs, the degrees of adjectives (e.g. “more”, “less”, etc.), the adverbs of mode (e.g. “completely”, “partially”, etc.), quantifiers (e.g. “very”, “little”, etc.);
- syntactic ones (e.g. the repetition of a phrasal structure that can produce a “cumulative” effect);
- rhetorical ones: metaphor, irony, hyperbole, etc.
- stylistic ones, e.g. through the emphasis (or, on the contrary, obscuring) of deictic reference to the I-here-now element can be identified in the contrast between *embrayage* and *débrayage*.

All these textual resources modulate their own possible semantic value of un-certainty by adapting to the dialogues of the specific context of enunciation in which they occur. Specifically, the broader framework of reference that we have adopted for understanding un-certainty is outlined by the notion of “social-epistemic rhetoric” (Berlin 1993), because it is considered responsive to the various research objectives and the typology of texts examined. In fact, by incorporating references both to the sociological tradition of the analysis of “ideologies” and to the semiotic investigation of “sign systems”, this construct allows to organize the complex variability of un-certainty markers in some patterns of global interpretation. The fruitfulness of this construct already springs from the sense of the word ‘rhetoric’, which evokes both the care for the word and the interests for the “style” – which is the link between linguistic and psychological (Caffi 2001). Furthermore, the reference to rhetoric involves connotations of functional “tools” to the specific purpose of the argumentation, even more than that persuasive character, in which the link with un-certainty plays a pivotal role.

Our approach tries to take into account the social context in which the argumentative activity takes place, as the participants are conditioned by certain values and by their knowledge: the scientific article is therefore valued in its belonging to a particular discourse “genre” (Bakhtin 1981). This concept identifies a set of texts characterized by a specific communicative function whose aims are shared by authors and users. In the specific case of our survey, the scientific article is not a “free” genre, but it is “packaged” to be accepted on the basis of criteria set by a specific scientific community. By its very nature, it is a discursive event with an argumentative intent and therefore, like all other argumentative texts, it is a “verbal” activity – inasmuch as it is configured in the context of textuality –, “social” – being the presence of argumentative subjects and of the public – “rational” indispensable – requiring a certain degree of reasonableness to be backed up (van Eemeren et al. 2002). On the other hand, its peculiarity is that it constitutes the privileged channel for the introduction of new discoveries in the scientific community. Indeed, its main purpose is to invite other scholars to take charge of the proposed “message”, to accept or defend a certain position, to accept any new knowledge produced (Hyland 2001).

## A Survey on the Evolution of Un-Certainty in Psychiatric Knowledge Texts

### Objectives and Research Questions

With regard to the context of enunciation under examination, the sources of diachronic variation in the discursive construction of un-certainty can be linked, on the one hand, to the different way in which the community of scholars intends the “body-mind” relationship; on the other, they are related to the different profiles of “authorship” emerging from the texts. Therefore, the fundamental questions underlying the present research study can be formulated as follows:

- what changes are traceable in the argumentative structure of un-certainty in the psychiatric scientific communication by the British Journal of Psychiatry in its life span? The attempts to answer this question will be based upon the search for linguistic and rhetorical devices that favour the construction of argumentative traces based on certainty and which, in a complementary manner, limit the applicability of acquired knowledge in the context of insane interactions between body and mind;
- how does the construction of socially “credible” authorship profiles evolve, in this scenario? This question is answered in order to gain validity in the public domain and acquires specific importance in the “scientific article” genre guaranteed and legitimized by the scientific community. Namely, each scientific community consists of a group of experts who represent the “ultimate judges” of the work itself. In return, the latter increases its accreditation as an institution able to validate the possible applications of the knowledge proposed to real life contexts. The authors’ argumentative proposal therefore represents a contribution within the dialogical space of the scientific community, which is responsible for sharing a worldview and a network of meanings. Therefore, in a complementary way, the evolution of the idea that authors cultivate with respect to the scientific community of reference will be explored.

### Interpretative Procedure

To enhance the dynamics of the discourse in its contexts, the texts of the articles were subject to a Critical Discourse Analysis (Fairclough 2003; Van Dijk 2008; Wodak and Meyer 2009), a perspective based on the understanding of language as social practice and argumentation as strictly anchored to the context in which it is produced. This indissoluble link involves a continuous and dynamic co-construction of reciprocal influences which, in turn, affect the situation, identity profiles, interpersonal relationships and public scenarios.

In the path of Critical Discourse Analysis that here will be referred to as “diatextual” (Manuti et al. 2017), the salient aspects of the texts and the related contexts of enunciation, referable to their “topic”, their “tenor”, their “key” (Halliday and Hasan 1985) are analysed. Therefore, changes in the tone of un-certainty must be due to the type of themes being dealt with, to the interlocutory relationship constructed through the text and to the “way” in which the positions taken are presented and argued.

The analysis of the articles was carried out through various interpretative apparatuses, by practicing both bottom-up and top-down approaches in order to perform a holistic reading of the texts (Mininni et al. 2014a). More specifically, in order to meet our research objectives, the bottom-up approach revealed the stylistic-rhetorical aspects concerning the discursive modulation through the pragmatic “mitigation” construct. Intended as a synonym of attenuation or weakening, mitigation is aimed at “the lowering of one of the parameters of interaction, for example the epistemic certainty, the accuracy of the propositional content, the intensity of the illocutionary force, the social role, professional competence, topic relevance, emotional salience, interrelated” (Caffi 2007, 6). By weakening one or more of these parameters, the mitigation involves a “reduction” of the interlocutors’ constraints (Meyer-Hermann and Weingarten 1982; Wunderlich 1976), so as allow for much more availability to reach the objectives of the interaction. This reduction can take place substantially on three levels, which can be illustrated through lots of metaphors. Each of them gives voice, with different strength, to the different attempts to “escape” the other through more or less “protective” shelters or through a more active willingness to conceal one’s position. You can mitigate discourse through (Caffi 2009):

- the reduction of the propositional content of the discourse, when the enunciator is repaired behind “bushes”;
- the attenuation of the illocutionary force of what has been said, when it is protected by “hedges”;
- keeping the distance from an actantial or space-time point of view, if it enjoys the confidentiality guaranteed by the “screens”.

Of course, mitigation also works in other ways, sometimes more complex to identify since they are configured as semantic or textual acts.

Looking at the texts in a top-down direction, even if they represent written texts, the articles are modelled as conversational events: they are proposed as a “voice” within the continuous dialogue aimed at building knowledge in a community of scholars. This is a testimony of how the “Principle of Protagoras”, revived by Billig (1987), according to which every sense proposition (*logoi*) stands in comparison with other positions (*antilogoi*), is also alive in scientific communication. Therefore, the “social-epistemic rhetoric” (Berlin 1993), which provides valid argumentative perspectives for particular sets of positioning, appears particularly relevant.

Halfway between bottom-up and top-down paths, it is possible to notice which “positioning” (stance) the enunciator can assume, with reference both to the nature of the information proposed by his/her textual world (Koustantoni 2004; Wesson and Pulford 2009) and to other analytical tools, concerning non-propositional aspects of discourse. In particular, through the expressions of comment and reformulation that act as “metadiscourse” (Crismore et al. 1993; Hyland 1996, 1998), enunciators can facilitate the reader not only the coherent organisation of the text, but also the development of his/her own personality and credibility. It is possible to draw a distinction between two forms of metadiscourse:

- textual metadiscourse, which allows to detect the intentions of the author for the use of “logical connectives” (e.g. ‘e’, ‘therefore’, ‘besides’, etc.), “frame markers” (e.g. ‘to finish’, ‘our objective here’, etc.), “endophoric markers” (e.g. ‘above mentioned’, ‘we will see it later’, etc.), the “obvious” ones (e.g. ‘X claims that...’, etc.) and “gloss practices” (e.g. ‘in other words’, etc.);
- interpersonal metadiscourse, which allows the enunciator to give a certain structure to his/her own relationship with the enunciator, thanks to the use of “attenuators” (e.g. ‘could’, ‘perhaps’, etc.), ‘intensifiers’ (e.g. ‘in reality’, ‘clearly’, etc.), “attitude markers” (e.g. ‘I agree’, etc.), “personal markers” (e.g. ‘me’, ‘our’) and “relationship markers” (e.g. ‘frankly’, etc.).

### The Study Corpus

In order to better organize the differences emerging at a diachronic level, the 160 years of the BJP life cycle have been divided into three periods, each lasting about five decades, also meeting criteria of more general historical coherence. The first period (1858–1900) presents the first constitutive steps of a psychiatric knowledge firmly rooted in the science/medical art. The second period (1901–1950) presents the progressive march of legitimization of the autonomy of psychiatric knowledge, also inspired by the confrontation with the emerging psychoanalytic theories. The third period (1951–2018) represents the development of the maximum interest for the social relevance of psychiatric knowledge as an interface between subjective well-being and public health. The last phase of this period (2000–2018) expresses the orientation to bring the understanding of mental disorders back into the orbit of cerebral functioning.

The corpus consists of 90 articles and represents a random selection – roughly one for each biennium – among those who, in the history of BJP, faced the “body-mind” connection (e.g. effects of psychotropic drugs, electroshock, behavioural manifestations connected to “organic” disorders). The corpus obtained in this way has been subdivided according to the belonging to one of the three above mentioned macro-periods: 1858–1900, 1901–1950, 1951–2018.

### Tracking Change

The changes detected in the discursive and argumentative construction of the tone of un-certainty can be traced back to two main axes of the organization of sense-making: the formal structure of the text and the conception of contents (psychic pathology and care practices). These two axes are intertwined to outline three different models of psychiatric knowledge that respectively represent the subjectivity of the researcher as “explorer”, “investigator” and “technician”.

The diachronic variation depends above all on the constraints of the discursive genre realized by the text. In fact it is precisely the way in which it changes over time to contribute decisively to modulating the tone of un-certainty with which the enunciator advances his/her own proposal. The scientific article begins to take shape in its enunciative peculiarity as a transformation of a type of original text consisting in the written elaboration of an “oral report”. In the first period, half of the selected articles are sorted out as “paper”, i.e. they are transcripts of oral presentations made by the authors in congress or association contexts. This percentage is reduced to a quarter in the second period and is completely absent in the third period. The evident traces of this original form are so important as to give the figure of psychiatrist the profile of an “explorer” of the intricate labyrinth of body-mind ties.

This intuition appears to be in line with the evolution, over time, of the same enunciative situations of meaning: in its initial phases, the BJP (as well as the other places of construction of scientific knowledge) has a “diffusive” (divulgative) function of events already ratified as “scientific” by a public discussion; in the final stages its (unique) function is “constitutive” of the public discussion on the issues of interest proper to the scientific community. In other words, at the beginning the BJP “manifests” the dominant orientations of the same community; later it “builds” them. This difference in the nature of the articles examined – papers vs texts – is only the most relevant indication of the changes occurred in the construction of scientific textuality. The evolution of the formal structure of the text has been considered by focusing the attention on two fundamental sections of the scientific article (Gross et al. 2002) – the introduction and the conclusion, because they have a decisive function both in terms of argumentation and of memorization. In particular, the argumentative nature of the scientific text appears to be salient in these sections for different and complementary reasons: the introduction has the function of presenting and framing the themes (also) through the synthesis of the relevant literature; the conclusion attempts to “close” the open problematization, by making a synthesis and drawing up the sums of what was previously exposed. More generally, these are two salient elements in the modern approach of the scientific article, founded on the acronym IMRD (Swales 1990) – Introduction, Method, Results, Discussion –, for their ability to accompany the reader and organize content, effectively making the complex scientific arguments easier to follow.

## Main Results

The preferred argumentative practices in different periods are affected by the efforts to anchor the discipline and the wider scientific community and, at the same time, by the building up of knowledge that each text proposes. Narrative, forms of *embrayage switching*, discursive modulations under the banner of mitigation, quoted expressions are resources that contribute to the identification of specific enunciation profiles, which are coupled with these anchoring and construction needs. The identified rhetoric, which collects the groupings of enunciative profiles, sees the researcher evolving between the explorer’s attempts at the investigator’s investigations and the critical rigor of the technician.

### The Un-Certainty of the “Explorer”

The socio-epistemic rhetoric of the un-certainty that frames the BJP textuality in the first period is marked by a “tendency towards personalization/dialogicity”. Especially when the texts are transcripts of oral accounts but also when presented according to the expectations of scientific articles, the modulation of un-certainty is regulated by an appeal to the intersubjectivity of knowledge. In fact, the argument uses strategies that are organized on the dialogic “I-you” or “I-all” scheme. The main strategies are:

1. personal involvement: e.g. “Accordingly I was appointed to visit the Turkish baths in England” (Baker 1889, 184);
2. the appeal directed to the reader: e.g. “If you will pardon me for quoting from myself” (Earle 1868, 374)
3. self-limitation: e.g. “We can only say that from our experience”, “I am able to record scarcely a single case” (Heimann 1887, 235)
4. the prevention of the reader’s objections: e.g. “I am painfully conscious of the objections that may be urged” (Clouston 1870, 28),
5. the *embrayage*: e.g. “I will here at present confine myself to the description of the following case” (Rorie 1862, 363),
6. the *debrayage* with appeal to the evidence – e.g. “It is within the experience of every medical”; “Experience leads me to the belief that” (Sheppard 1867, 65, 71) – or to “common knowledge”. The tone of certainty that resounds in evoking shared knowledge can derive both from the common sense – e.g. “Become widely recognized” (Robertson 1884, 54) – that from the specific background of their “community of practices”, i.e. psychiatrists: e.g. “As is well-known to experimenters [...] it is held by many, if not all, physiologists” (Diarmid 1876, 21).

Naturally, the aforementioned modulations of the tone of un-certainty can variously intertwine with one another, as in Extract 1 which, while valuing the experience as the basis of a given enunciative positioning, recognizes its scarce inferential power.

- Example 1: “My observations in this sphere, as yet, are too few to warrant the deduction of any general conclusions” (Robertson 1884, 55).

Recourse to the metadiscursive plan does not record any particular differentiation, since both relational focal resources are valued, e.g. “The circumstance, frankly admitted, that we...” (Müller 1880, 65), and resources with textual focus, e.g. the organizing list, as in “I would speak first of the use of the Turkish bath in the treatment of the insane as a curative agent, and secondly as a palliative agent” (Baker 1889, 185).

Taking a closer look at the different sections of the articles, in the opening words the construction of the phrases mainly aims to attract the attention and curiosity of the interlocutor, through the following strategies of enunciative modulation of the argumentation:

- use of the first person, both singular and plural, and / or the expression of one’s own point of view (e.g. “In presenting my views related to the medical treatment of insanity I will be very brief, tracing only the general outlines of the course pursued by me in a few of the best-marked forms of this disease”, Ranney 1858, 450) to support “subjective” verbs with emotional valence (“I am glad to have an opportunity of laying my experience in this matter”, Meyer 1896, 261);
- recourse to the “metadiscursive” enunciative structure with the prevalent option of relational metadiscursive markers;
- precise spatio-temporal references;
- great use of affective markers, in the form of positively and negatively connoted qualifying adjectives (e.g. important, excellent, prominent vs. unsatisfactory, scandalous).

In other words, the arguments put forward at the beginning of the texts aim at establishing with the reader a contract of communication inspired by interpersonal conversation, thus enhancing an “oral-literary” clause. Therefore, the tone of certainty is modulated by reference to personal experience, tradition and common sense. On the other hand, the various forms of mitigation present in the ambivalent and complex arguments bring out the caution with which to accept the proposed themes. In line with the general approach of the scientific article in this period, the conclusions also present aspects that proceed in this “narrative” direction through metaphorical and literary clauses (e.g. “this drug ought to be rescued from the oblivion into which it has so undeservedly and unaccountably fallen”, Hills 1874, 422) as well as reference to the “happy ending”.

- Example 2: “Believed from these distressing symptoms, the patient soon began to occupy himself industrially, and is now quite convalescent, having to a great extent recovered his former cheerfulness, to which he has been a stranger for eleven years and a half” (Rorie 1862, 365).

Between personal experience and narrative dimension, between timid impulses of certainty and more widespread acknowledgment of un-certainty, the articles end many times evoking a particular aspect of emotional resonance: hope. This appeal seems to go beyond a strictly personal dimension and appears to be an attempt to involve the community of reference. This implies that the research horizon in the scientific area of psychiatric pathology not only proposes “cold” data, but shows a marked “human” inclination and a stimulus to the future: for instance, “We hope that hypnotism will be given” (Smith and Myers 1890, 213); “The most hope of improvement” (Telford-Smith 1895, 289).

The modulation of the tone of un-certitude is often entrusted to the “reported discourse”, reported by the quotation marks and operating under both transparency and opacity (e.g. “and other such-like “secrets”, Sheppard 1867, 69). Indeed its most important peculiarity consists in giving consistency to the widespread practice of quotations, which thus assume a strongly dialogical value. In this period, the content of the “quotation marks” is constructed in a discursive process of sense-making that assimilates it to philosophical reflections, moral maxims or scientific laws.

The continuous presence of the voice of the other (whether he is a colleague or a patient), the continuous confrontation with his own position, the addition of comments relating to the voices of others, the possible juxtaposition between the voice of the other and his own voice, they contribute to creating a dynamic and dialogical sense of knowledge building. This multiplicity of voices ends up representing the veracity of what was said in a way that aims to simulate orality and sometimes even to “incorporate” it:

- Example 3: “After twenty-five minutes, in answer to the remark, “Now you are asleep” she quickly said, “No, I am not; I’m conscious, that’s all I know”. She began to laugh suddenly, after half-an-hour, and remarked, “I had this done to me before by a lady”, then suddenly shouted, “I hate Nelly Farren; I hate her photograph, Jack Shep herd and all” (Smith and Myers 1890, 209).

The strategy of dialogism is considered so compelling that sometimes authors go so far as to set up fictitious dialogues, imagining the answers aimed at explaining the reaction of others to their theses. When a dialectical debate is set up, a “sequential strategy of manifestation of disagreement” is mostly used (Caffi 2007), but the continuous reference to the enunciative axis of the metadiscourse and of the narration of a story anchors the text to a tone of un-certainty elaborated in a regime of familiarity (“our author”).

### The Un-Certainty of the “Investigator”

The socio-systemic rhetoric of the un-certainty practiced in the second period outlines the psychiatrist profile as an “investigator”: it is a form of authorship that in some respects recalls the one of the first group, in its “first-person” and narrative mode. For others, however, some innovations are found both from a structural and rhetorical point of view: the text appears to be based on criteria of greater “objectivity” and accompanied by the tendency to “problematize” existing issues, rather than to argue according to “one’s own word” or what is “evident”. Therefore, the tone of un-certainty derives from the progress of the text due to problematization and comparison of positions based on “sequential strategies of manifestation of disagreement” (Caffi 2009), as in examples 4 and 5:

- Example 4: “Schmidt’s method should be noted [….] Salkowski’s method is the best” (Porter Phillips 1910, 428).
- Example 5: “Academic psychologists agree […] Clinical psychiatrists do not” (Monro 1950, 261).

On several occasions, we tend to value the “double soul” of the “object” of psychiatric research, because the problems faced capture, sometimes within the same sentence, physical and mental aspects of medical and psychological relevance.

If in the first period the reader is involved through first-person discourse, narratives and metadiscursive formulas, in this new context of enunciation formulas are used that perform this function by recalling the sense of responsibility and opportunity of the textual proposal: “the present seems to be an opportune moment for reviewing what has been done [...] it is important to remember that [...]” (McAlister 1925, 236).

Also with respect to the argumentative structure of the opening and closing periods of the articles published between 1901 and 1950, elements of continuity and discontinuity with respect to the first period are found. For example, the incipits are characterized by a rhetoric of problematization, which aims to involve the reader in the heart of the events, since s/he recognizes a greater “competence”.

The concluding remarks, on the other hand, although they often re-evoke “hope”, seem to exhibit a greater enunciative “maturity”. In fact, the “final” sometimes tends to move away from the content to propose a sort of “ethical contract” in the construction of a particular scientific community. The latter seems to represent not so much the source of validation of the position taken, as a column to which to lean against the widespread un-certainty of which, in this period, the authors of the articles seem to be aware. The un-certainty transpires from the swing between limitations and auspices, from the recognition of both the doubts and the possible benefits produced by pharmacological treatments on the mind and, more generally, by the clear awareness of the complexity of the mind-body nexus.

Also with reference to the citations, the second period presents elements of continuity with the first one. For example, some quotes offer aphorisms and maxims (e.g. “First of all, I think of Sir William Gull’s dictum to him many years ago: ‘Remember that the brain is a gentleman with many servants [...]’”, Raw 1902, 25), others offer literary quotations (“In ‘Romeo and Juliet’ Shakespeare makes Mercutio ridicule”, “music with her silver sound”, Mott 1921, 170) or paraphrase of the same (“To paraphrase Leibnitz, ‘There is nothing in the image that was not first in the percept’”, Hutton and Bassett 1948, 336).

We also find a narrative format aimed at involving the scientific community: to make the reader feel directly involved, the authors tend to build stories, alternating moments of description even meticulously, with emphasis on details (e.g. “Three long breaths and three sighs, together with the correct mouth formation, produces the sentence ‘How are you?’”, McDowall 1918, 63) in dialogues that report direct discourse sequences. The enunciative situation in which the reader is involved encourages, therefore, the attention and interest in the evolution of the case.

In the second half of the fifty-year period, there is a tendency to reduce citations to increase quoted expressions based on transparency and opacity. In particular, alongside the focus on headwords to clarify the meaning (e.g. “The term ‘psychoses’ therefore, is here used in its broadest sense”, Molony 1927, 66), the use of quotation marks is aimed at presenting discourses with technical and disciplinary traits, strictly psychiatric vocabulary, concepts, theories. In this period, therefore, dialogism is configured as “problematic”, with the aim of categorizing, classifying, comparing (“This constitutes the ‘indirect somatic response’. Secondly, it may flow cortically to form a ‘viscero-cortical’ or ‘viscero-thalamic’ response”, Harris 1934, 487) or building knowledge through textbookish explanations.

In short, the use of quotations and quotation marks testifies to the ambivalence of this transitional period: on the one hand, it supports the enunciation of the “psychiatrist narrator” and the construction of a dialogue based on direct discourse and on the first person “singular”; on the other, it enhances the first “plural” person, operated by a psychiatrist addressed to the scientific community. The latter is included both in the problematization of the issues and in the form of dialogism based on the sharing of technical and scientific arguments. In other words, citations no longer have the sole function of “validating the story told”, but at the same time they pursue the aim of building a community of practice (Lave and Wenger 1991) in which the “shared repertoire” is realized through a shared sociolect and its effective interpretation. Indeed, in addition to explaining concepts and passages, it is also how the words are understood, metadiscursively speaking. It is therefore a narrator who, while maintaining his/her own polyphony, is ethically involved in the construction of a scientific community.

### The Un-Certainty of the “Expert”

The rhetoric that expresses the positioning of the third period certifies, as occurred in the second period, the coexistence of two “souls”: the emerging profile of the psychiatrist is that of an “antidogmatic expert”. On the one hand, there is a tendency towards greater rigor, through marked precision and punctuality in the description of the administration of the drug, attempts to “quantify” some characteristics of the disease, as well as through meticulous explanation of the methodologies used (e.g. 6).

- Example 6: “Only patients who had a mean psychosis score of four or less (mean 2.0±0.3, range 1.0 to 4.0), which indicates absence of or some residual psychotic symptoms and is considered to be non-psychotic on our unit, and who had a mean depression score of four or more were included (mean 6.5, range 4-8.3)” (Tómasson 1936, 602).

On the other hand, aspects that detect the presence of a “technical” psychiatrist are balanced by the definitions of the self as “anti-dogmatic”. It is during this period, and in particular in 2004, that the paragraph dedicated to the presentation of the “limits” – “Strengths and limitations of the study” – appears in the structure of the text; in 2010 this space would be explicitly labeled as “Limitations”. Nevertheless, “bushes” and “hedges” have been operating since the early years of the third period to “mitigate” certainty in the presentation of body-mind themes. In particular, both through the use of metadiscourse (e.g. “It would seem, therefore, that”, “not only [...] but also [...]” “However, in other cases [...]”) and through a widespread tendency to contextualize what has been said (e.g. “In other cases [...]” “In the cases with […]” “Here the difference is [...]” “In some cases […]”), the discursive and argumentative strategies limit the scope of their assertions. These indications are accompanied by discursive acts and rhetorical strategies based on “caution” (e.g. 7):

- Example 7: “The fact that if psychogenic factors are present the outcome is rather better than otherwise, both in the treated and untreated series, should be only cautiously applied clinically” (Polonio and Slater 1954, 444).

In a more homogeneous and clear way compared to the other periods, the incipit of the articles concerns the topic of reference, immediately using a technical and scientific lexicon and citing the previous literature from the outset, by emphasizing in particular the results of the research. Indeed, the function of the principle seems to be to synthesize the results previously obtained, illustrating positive aspects (“value”, “importance”) and innovative aspects, as well as the limits of the same, thus creating a space for one’s work in a frame consisting of a careful problematization of the issues illustrated; on the other hand, the author, thanks to the coexistence of arguments and counterarguments, acquires greater authority and credibility.

The incipits of the articles of this period assume an almost standardized structure: the first measure assumes an assertive-descriptive tone, as in the example “Electroconvulsive therapy (ECT) is useful in the treatment of several psychiatric syndromes, most noticeably major depression. It is also the treatment of choice in manic excitement not responding to chemotherapy (Taylor 1982). In addition...” (Atre-Vaidya and Jampala 1988, 55). On the other hand, although structured as assertive in vocabulary and syntax, they are limited in their illocutionary force by means of conjunctions, adjectives and adverbs, presentation of the limits of current research, argumentation for “exception” (“DESPITE the good results [...] Nevertheless, the overall situation”, Quinn et al. 1960 160). These mitigation proposals produce, as a general effect, the support of problematization and the complexity of the scenario, sometimes explicitly explained, especially when the intersection between physical and mental, scientific and moral aspects is touched (e.g. 8):

- Example 8: “The management of sexual delinquents, especially those with compulsive putting into action of aggressive sexual impulses, poses great problems for the medical profession from the therapeutic as well as the moral standpoint” (Cooper et al. 1972, 59).

In respect of empty arguments related to doubts, lack of certainty or positions, one’s work inevitably appears as “opportune”, a useful attempt to provide elements for further clarification and reflection (e.g. 9):

- Example. 9: “little is known about [...] Population-based samples, followed longitudinally, may be helpful” (Colman et al. 2008, 327).

The proposal of one’s research can also be accompanied by metadiscursive reflections, suspending the formality of impersonal discourses for the benefit of expressions characterized by embrayage, as in the example “We felt that it would be important to clarify these issues” (Roeder and Heshe 1976, 241).

With reference to the conclusions, the formula “the results suggest ...” is very recurrent, emblem of the transition from the first to the third person, from the attempts to build the knowledge based on personal experience and on what is known to promote the contribution of the search. Emblematic, compared to this evolution, is the passage from the 1950 text, in which conclusions display formulas such as “I do not suggest [...] I submit, however [...] I suggest that [...] I formed the clinical impression that [...] I submit, therefore” (Monro 1950, p. 263), to article of 1952, in which it is possible to read that “The results obtained suggest [...] The most satisfactory results [...] equally marked beneficial results” (Smith 1952, 163).

Alongside the change in verbs and personal / impersonal formulas, adjectives also adapt to the new way of constructing knowledge, which is now more technical and objective (e.g. ‘effective’, ‘satisfactory’, ‘marked’, ‘defined’). In the attempts to build certainty, based on statistical significance and agreement with the literature, there are many elements of un-certainty, until paradoxically to re-propose the use of “old methods” despite the “sufficiently encouraging” results of research (example 10).

- Example 10: Thus, while the results reported here and those of previous studies are sufficiently encouraging to repay further inquiry, they should not lead to the abandonment of the standard method which is well-tried and effective (Levy 1968, 462).

In the presentation of the results there is also space for “negative” results as in the example “The view that patients [...] was not supported in this study. The concern that [...] appear to be justified” (O’Leary 1996, 327) and the importance that even the negative results or the lack of results may have with respect to the progress or otherwise of knowledge.

Consequently, in the final stages of the articles there is also space for future job prospects: we pass from “hope”, which permeated and animated the conclusions of the first and second period, to the “desirable”, “imperative” and “dutiful” work that should be implemented. From this point of view, it seems that the proposal for future work represents an invitation to the community to accept what was presented, even if “uncertain” and “limited”, and to keep the attention on the topic high, so as not to close the proposal contract to the reader.

In the third period the quoted expressions are less marked by direct quotations and used more frequently as deictic deictic screens (Caffi 2009). The author appears to be meticulously oriented towards the technical-scientific description of the work, but also projected towards a “mitigated” construction of the discourse. In this frame, the “literary” quotations are drastically reduced and those of a technical-scientific nature, of a more exquisitely psychiatric nature, become a sign of a scientific community considered more “mature” and “aware”. The alternative positions are not explicitly mentioned, but must be “rediscovered” by a comparison of the sources reported in the notes. Therefore, direct quotations (indicated by the use of quotation marks) – both by other scholars that share their position and by those who oppose and / or differentiate their opinion – are almost completely absent. In some respects, this textual construction strategy increases the impression of certainty, because the argument proceeds assertively.

Being less oriented on the citational side, quoted expressions are more clearly subdivided into “opaque” and “transparent”. In particular, whereas the transparent ones are anticipated by an announcement (“This concept of ‘supersensitivity psychosis’”, Peet and Collier 1990, 581), the opaque ones correspond to the deictic screens of “so to speak...” (“At the time of admission to our hospital, she was receiving” carbamazepine (400 mg daily) and chlorpromazine ‘as needed’”, Atre-Vaidya and Jampala 1988, 152). Confirming an essential argumentative function already emerged in the second period, the use of quoted expressions has the function of classifying, categorizing and comparing, proposing a dialogical form based on complexity. Moreover, the voices of the others continue to be present, not only in the “real” version of the patient – reporting the words as in previous periods – but also “ideal”, imagining or generalizing responses through the proposal of temporal and active space screens (ex. 11).

- Example 11: “Sometimes somatic features, e.g. sweating (as in Case I) represented the main factor disturbing the patient, and became so prominent that projection ultimately gave rise to the sequelae of ideas of reference – i.e. ‘people talk about the way I smell’” (Smith 1952, 163).

By proposing an extremely concise vision of the forms of dialogism, we move from a dialogism based on interpersonal relationships to a form of dialogism aimed at building a scientific and linguistic community, a task that seems “outdated” in the third period, in which we go back to a more competent and aware community.

## Discussion of Findings and Concluding Remarks

The theoretical considerations that have guided the epistemological and methodological approach of the present study allow us to propose a modelling on the communication of un-certainty in the psychiatric scientific domain based on linguistic, discursive and argumentative aspects. According to this model, the sense system of un-certainty emerges from the tuning of enunciative resources detectable on three levels:

A. *contextual scaffolding*: references to the discursive genre (such as citations, notes, etc.) and to intra- and intertextual dynamics;
B. *enunciative modulation*: mitigation ripples, multimodal resources of vagueness, argumentative strategies (cancellation of the ego, etc.) and stylistic options for various reasons;
C. *textual sequence* (lexical and syntactic markers).

This complex interpretative apparatus converges in the definition of three socio-epistemic rhetoric, whose value can be fully learned in their configuration to the interweaving of content and style-rhetorical aspects, between proposals of researchers’ positions and attempts to build-validate the community scientific of reference, within the genre for the construction of scientific knowledge *par excellence*: the article.

Looking at the articles in their entirety, the texts of the BJP evolve towards the involvement of the scientific community less and less based on the personal experience of the author and increasingly oriented on an argument based on the presentation of research contributions. In particular, the evolutionary excursus makes it possible to identify a succession of stylistic options that lead ever more clearly to the “erasing of the Ego” of the text enunciator. The empirical parts presented, albeit rigorous and precise (as evidenced by the assertiveness and objectivity in the presentation of research and results, as well as the support of graphs, tables and numbers), are affected by the limits of the variety of human experience and countless aspects that involve the body-mind relationship and that make the issues always addressed “problematizable” and contextualised.

The “life cycle” of the construction of un-certainty in the BJP magazine describes the evolution from an “assertive” perspective, which is not typical of the scientific tradition but mostly close to the narrative genre – cultivated by the guarantee of experience, of the “common knowledge” and from the pretence of validity through embrayage – to a more “assertive” proposal from the point of view of methodological construction, but equally “critical”, as closer to the scientific tradition and the problematic of the issues related to the body-mind relationship. In addressing the problems of the un-certainty of the contents expressed, the authors emphasize particular expressive modalities and argumentative strategies functional to the types of texts they propose.

From a rhetorical point of view, one of the most interesting results of our investigation concerns the progressive disappearance of the “appeal to the mystery” as a basis for the un-certainty of psychiatric knowledge. In the first century of its constitution it is possible to come across statements such as “The treatment of insanity is the great desideratum” (Ranney 1858,450) or “How the results are obtained is something of a mystery” (McAlister 1925, 236). This resource of meaning authorizes us to believe that the modulation of the tone of un-certainty stems from a widespread awareness of crossing an “unknown land”, linked to the issues of psychic pathology.

Within such changing scenarios, a look at the different sections of the texts makes it possible to detect the salient argumentative traces. The incipit of a scientific article has the fundamental task of overcoming the reader’s skepticism, capturing his/her interest and trying to encourage him/her to continue reading. In the case of our corpus, this proposal seems to evolve from forms of “personal recall” to forms of involvement of the reader as a member of a scientific community, activating resources, knowledge and skills and involving them in a complex and problematic reality. Whether it is to illustrate the organization of work or to summarize the literature and the positions of scholars on the subject, the author shows his/her “model reader”, initially proposed as an empathic “auditor” of a story, gradually promoted to a member of the scientific community, thus accompanying him/her in reading the text.

Contrary to expectations, in the analyzed texts conclusions do not have the function of “closing” the proposal of the communication in the article, nor of “sedation” of doubts and biomedical-psychiatric problems; rather, they represent a “den of un-certainty”, made up of subjective reflections, partial and contextual results, enunciative mitigation and leaps towards the future. In this sense, the authors, aware of a proposal for the construction of less “certain” knowledge, propose contracts that go beyond the text in question: they consider not so much the intratextual dimension (to “close” the article by recalling the way in which has been opened), but take into account its intertextual (recalling the literature or their future works) and interdisciplinary characteristics. In particular, the reference to the (also) ethic responsibility of the disciplines to work together appears to be salient. It seems that the contract proposal based on certainty is postponed to an “elsewhere” dimension, re-launching in the reader the hope and the need for new knowledge, in the current awareness that, in the context of psychiatric scientific communication, certainty can wait.
